# Supplementary material for: Identification of photoperiod-induced specific miRNAs in the adrenal glands of Sunite sheep (Ovis aries)
Source: Front Vet Sci. 2022 Jul 22;9:888207. doi: 10.3389/fvets.2022.888207 (PMC9354845; doi:10.3389/fvets.2022.888207)
Supplement: Supplementary file 1 [file Data_Sheet_1.ZIP › Supplementary file/miRDeep2 file.zip!/miRDeep2 file/Know_Structure/SPLP42A1/oar-mir-133.pdf]

miRBase precursor : oar-mir-133
Total read count : 915
oar-miR-133 read count : 890
remaining reads : 25

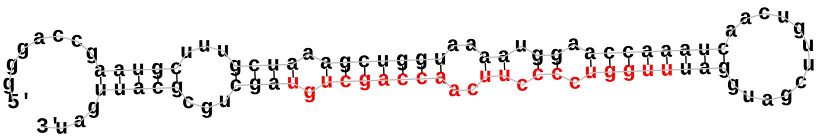

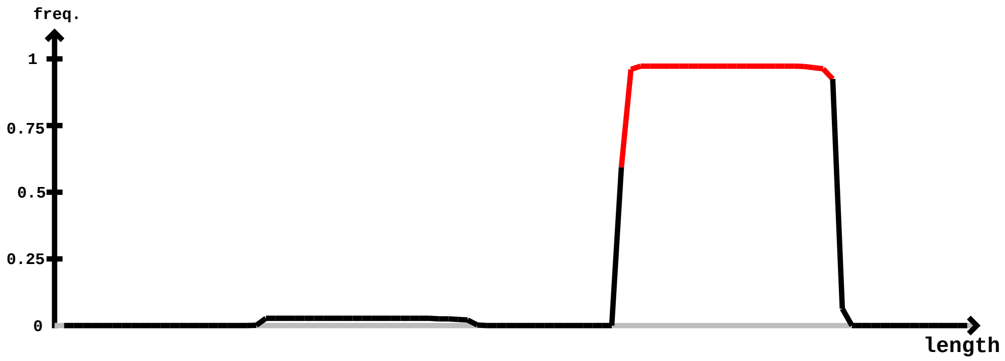


oar-miR-133

```
5'- gggaccgaaugcuuugcuaaagcugguaaaauggaaccaaaucaacuguucgauggauuugguccccuucaaccagcuguagcugcgcauugau   -3'    exp
    .......(((((...((((.(((((((..((.((.((((((((............)))))))).)).))..))))))).))))...)))))...    reads      mm         sample
    ...................Gagcugguaaaauggaaccaaau...................................................    1          1          seq
    ....................agcugguaaaauggaacc.......................................................    2          0          seq
    ....................agcugguaaaauggaaccaa.....................................................    2          0          seq
    ....................agcugguaaaauggaaccaaa....................................................    2          0          seq
    ....................agcugguaaaauggaaccaaau...................................................    16         0          seq
    ....................agcugguaaaauggaaccaaauc..................................................    2          0          seq
    .........................................................uuugguccccuucaaccag.................    1          0          seq
    .........................................................uuugguccccuucaaccagc................    3          0          seq
    .........................................................uuugguccccuucaaccagcu...............    3          0          seq
    .........................................................uuugguccccuucaaccagcug..............    28         0          seq
    .........................................................uuugguccccuucaaccagcuA..............    6          1          seq
    .........................................................uuugguccccuucaaccagcugu.............    482        0          seq
    .........................................................uuugguccccuucaaccagcugua............    20         0          seq
    ..........................................................uugguccccuucaaccagc................    1          0          seq
    ..........................................................uugguccccuucaaccagcu...............    1          0          seq
    ..........................................................uugguccccuucaaccagcug..............    1          0          seq
    ..........................................................uugguccccuucaaccagcugu.............    295        0          seq
    ..........................................................uugguccccuucaaccagcugua............    38         0          seq
    ...........................................................ugguccccuucaaccagcugu.............    11         0          seq
```
